# Oral β-Lactam Pairs for the Treatment of *Mycobacterium avium* Complex Pulmonary Disease

**DOI:** 10.1093/infdis/jiad591

**Published:** 2023-12-27

**Authors:** Dereje A Negatu, Sung Jae Shin, Su-Young Kim, Byung Woo Jhun, Véronique Dartois, Thomas Dick

**Affiliations:** Center for Discovery and Innovation, Hackensack Meridian Health, Nutley, New Jersey, USA; Center for Innovative Drug Development and Therapeutic Trials for Africa, Addis Ababa University, Addis Ababa, Ethiopia; Department of Microbiology, Graduate School of Medical Science, Brain Korea 21 Project, Yonsei University College of Medicine, Seoul, South Korea; Division of Pulmonary and Critical Care Medicine, Department of Medicine, Samsung Medical Center, Sungkyunkwan University School of Medicine, Seoul, South Korea; Division of Pulmonary and Critical Care Medicine, Department of Medicine, Samsung Medical Center, Sungkyunkwan University School of Medicine, Seoul, South Korea; Center for Discovery and Innovation, Hackensack Meridian Health, Nutley, New Jersey, USA; Department of Medical Sciences, Hackensack Meridian School of Medicine, Nutley, New Jersey, USA; Center for Discovery and Innovation, Hackensack Meridian Health, Nutley, New Jersey, USA; Department of Medical Sciences, Hackensack Meridian School of Medicine, Nutley, New Jersey, USA; Department of Microbiology and Immunology, Georgetown University, Washington, District of Columbia, USA

**Keywords:** nontuberculous mycobacteria, *Mycobacterium avium* complex, β-lactam, synergy, target attainment, tebipenem, sulopenem, amoxicillin, cefuroxime

## Abstract

Cure rates for pulmonary disease caused by the *Mycobacterium avium* complex (MAC) are poor. While β-lactam are front line antibiotics against *Mycobacterium abscessus* pulmonary disease, they have not been used or recommended to treat MAC lung infections. Through a comprehensive screen of oral β-lactams, we have discovered that selected pairs combining either a penem/carbapenem or penicillin with a cephalosporin are strongly bactericidal at clinically achieved concentrations. These dual β-lactam combinations include tebipenem and sulopenem, both in phase 3, and Food and Drug Administration-approved amoxicillin and cefuroxime. They could therefore immediately enter clinical trials or clinical practice.

## Graphical Abstract

**Graphical Abstract:**
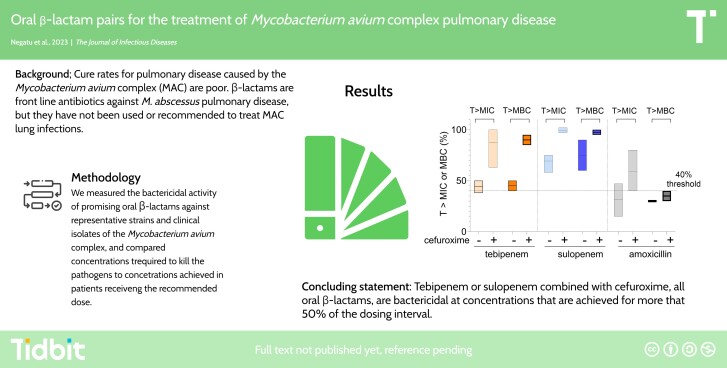

## Teaser

Oral β-lactam pairs combining tebipenem, sulopenem, or amoxicillin with cefuroxime achieve strong bactericidal activity at recommended doses against pulmonary disease caused by the *Mycobacterium avium* complex.

Globally, the incidence and prevalence of nontuberculous mycobacterial (NTM) infection and pulmonary disease (PD), caused by a collection of environmental mycobacterial species, have been on the rise for several decades. The dominant group of NTM pathogens worldwide is the *Mycobacterium avium* complex (MAC), which includes *M. avium* and *Mycobacterium intracellulare* as the clinically most relevant etiologic organisms [1]. Persons with cystic fibrosis, non-cystic fibrosis bronchiectasis patients, and individuals with a weakened immune response are all at increased risk of developing NTM-PD infections.

Treatment of MAC-PD calls for multidrug therapy with a macrolide (clarithromycin or azithromycin), rifampicin or rifabutin, and ethambutol [2], and mean treatment duration generally exceeds 1 year. Amikacin, an injectable or inhaled aminoglycoside, is added to treat cavitary disease, with hearing loss as a frequent side effect. Despite such intensive treatment, favorable outcomes of 40% to 70% are reported [3]. In patients with either cavitary or macrolide-resistant PD, mean treatment duration often doubles and 5-year mortality rates as high as 25% to 50% have been reported [4]. Not surprisingly, the loss of macrolide susceptibility is a major driver of poor prognosis in MAC-PD because a substantial portion of the minimum inhibitory concentration (MIC) distributions of rifampicin, rifabutin, and ethambutol against MAC exceeds evidence-based clinical breakpoints [5]. In contrast, the MIC distributions of clarithromycin against macrolide-susceptible MAC isolates are mostly below the clinical breakpoints [5]. Macrolides are, however, bacteriostatic against MAC at clinically achieved concentrations, which may limit their utility in immune-compromised patients where antimicrobial therapy is poorly assisted by the immune system, and at sites of chronic infection where quiescent bacterial populations persist [6]. In vitro studies in a hollow-fiber system suggest lack of efficacy by the first-line regimen clarithromycin-rifampicin-ethambutol [7].

More potent, bactericidal, oral, and well-tolerated antibiotics are urgently needed to improve these underachieving regimens. Repurposing of approved drugs and late clinical development candidates constitutes a pragmatic approach to deliver short-term results. While cefoxitin and imipenem, 2 injectable β-lactams, are recommended for the treatment of *Mycobacterium abscessus* PD [2], β-lactams are not considered against MAC-PD [1]. The 3 classes of β-lactams, penicillin, cephalosporins, and penems/carbapenems, have an excellent safety profile, are often bactericidal around their MIC [8], and a growing number of orally bioavailable β-lactams are in clinical use or late clinical development. The combination of ceftazidime, an injected cephalosporin, with parenteral β-lactamase inhibitor avibactam, has shown promising results in the hollow-fiber system, but whether adequate pharmacokinetic-pharmacodynamic (PK-PD) targets can be achieved at the clinically approved dose remains to be assessed. A targeted screening of 4 parenteral cephalosporins (cefoxitin, cefoperazone, cefmetazole, and cefepime) revealed selective activity against *M. intracellulare* but not *M. avium* [9, 10]. In a study from the 1980s, a larger panel of β-lactams was profiled against 30 MAC clinical isolates from persons with human immunodeficiency virus (HIV), showing lack of relevant activity except for amoxicillin and imipenem against a small subset of the strains [11]. However, systematic screening of oral penicillins, cephalosporins, and carbapenems against the major MAC species has not been reported.

In previous work, we have uncovered strongly synergistic and bactericidal oral β-lactam pairs against *M. abscessus* [8], consistent with emerging insights into the molecular basis of their mechanism of action, suggesting that judicious pairing of β-lactams can overcome the redundancy of peptidoglycan biosynthesis enzymes [12]. Here we have extended our approach to *M. avium* and *M. intracellulare* to assess the potential of oral β-lactams for the treatment of MAC-PD and discover β-lactam pairs with bactericidal activity at clinically achieved concentrations. Synergies in growth inhibition do not always translate from bench to bedside, for microbiological and pharmacological reasons. To avoid classic in vitro-in vivo disconnects, (1) we screened for synergies between β-lactams, thus minimizing PK mismatch, (2) we confirmed that synergistic pairs in growth inhibition also achieve bactericidal synergy, and (3) we retained only β-lactam pairs for which the MIC and minimum bactericidal concentration (MBC) lie within the clinical breakpoints established for other pulmonary infections and for which the PK-PD target of free time above MIC (*f*T > MIC) > 40% is met.

## METHODS

For single-point screen and MIC determination, exponentially growing cultures (with an initial optical density at 600 nm [OD_600_] of 0.4 to 0.8) were adjusted to 10^5^ colony-forming unit (CFU)/mL in Middlebrook 7H9 broth and seeded onto 96-well plates containing 10 μM of each study drug (single point) or serial dilutions (MIC) as indicated, to a final volume of 200 μL/well. The plates were incubated at 37°C for 7 days at 110 rpm. Growth was monitored by absorbance at 600 nm, and percent inhibition was calculated relative to the untreated controls. MBCs were determined by plating on Middlebrook 7H10 medium for CFU enumeration of drug-treated wells compared to starting inoculum. In growth inhibition synergy experiments, the standard checkerboard titration assay was used as described previously [8].

## RESULTS

**Figure 1.**
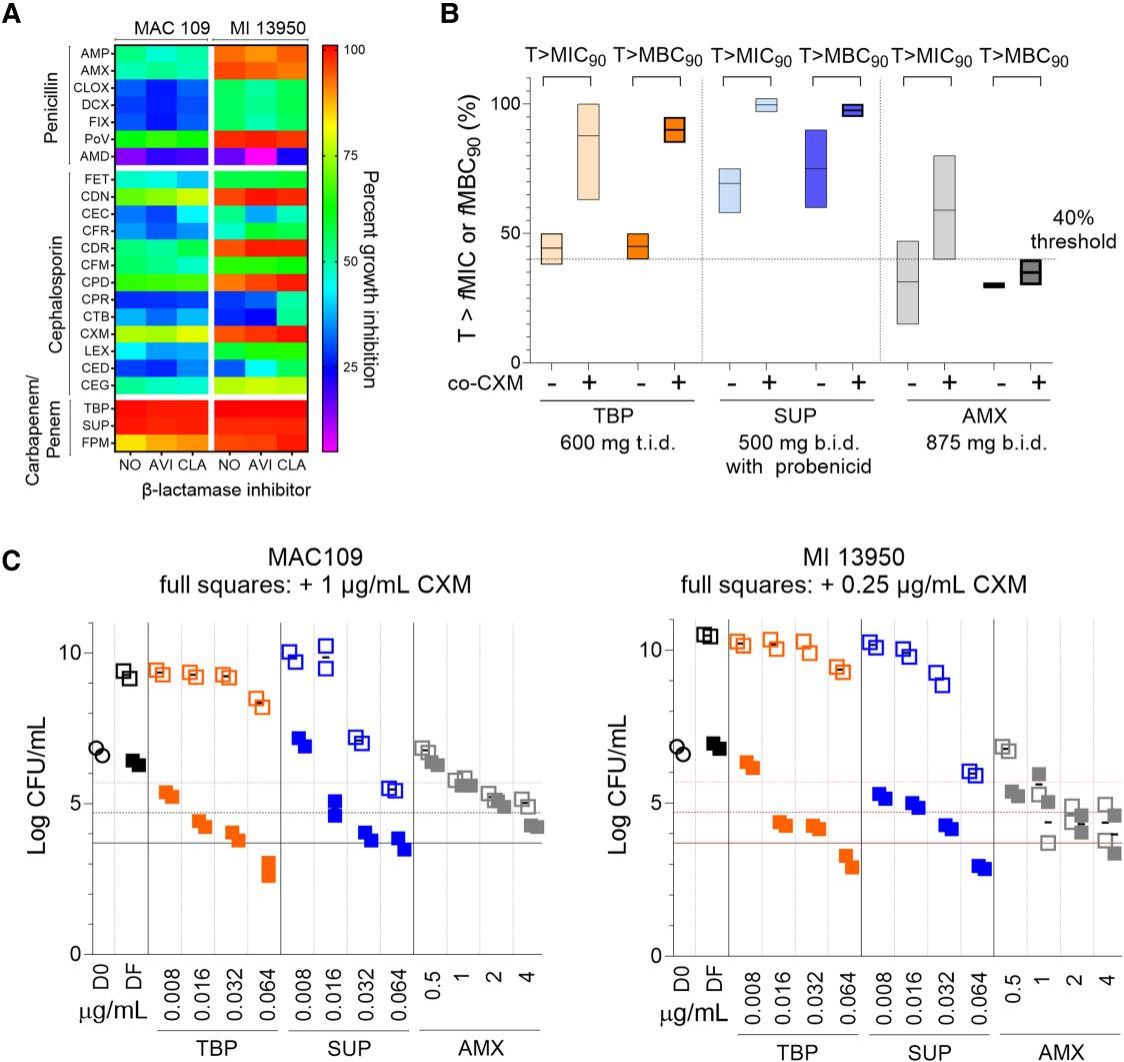
Identification of oral β-lactam combinations achieving standard pharmacokinetic-pharmacodynamic targets against MAC pulmonary disease. *A*, Growth inhibition of *M. avium* subsp. *hominissuis* MAC109 (MAC109) and *M. intracellulare* ATCC13950 (MI13950) reference strains by oral β-lactams (10 µM, 3.5 to 5.5 µg/mL) with and without β-lactamase inhibitor AVI (4 µg/mL) or CLA (2.5 µg/mL). TBP, SUP, AMX, and CXM were selected for subsequent experiments. Drug characteristics are provided in Supplementary Table 1. *B*, Fraction of the dosing interval during which plasma concentrations were above the MIC_90_ or MBC_90_ (concentration that inhibits growth by 90% or kills 90% of the bacterial population, respectively) for the most potent single and dual β-lactams against MAC109 and MI13950 reference strains, corrected for protein binding. Clinical pharmacokinetics data from healthy volunteers were retrieved at doses and dosing frequency used in phase 3 trials for TBP and SUP, and at doses used for the treatment of lower respiratory tract infections for AMX. SUP is dosed twice daily with coadministration of probenecid to reduce renal clearance, and TBP is dosed 3 times daily. *C*, Dose-response bactericidal activity of the most potent single and dual β-lactams against MAC109 and MI13950 reference strains showing strong bactericidal synergy when cefuroxime is added to either TBP or SUP (filled squares) compared to TBP or SUP alone (empty squares). CXM was added at a fixed subinhibitory concentration of 1 μg/mL against MAC109 and 0.25 μg/mL against MI13590 because its MIC when used alone is < 0.5 μg/mL. CFUs were enumerated by plating on Middlebrook 7H10 agar medium. Abbreviations: AMD, amdinocillin; AMX, amoxicillin; ATCC, American Type Culture Collection; AVI, avibactam; CDN, cefditoren; CDR, cefdinir; CEC, cefador; CED, cefradine; CEG, cefaloglycin; CFM, cefepime; CFR, cefadroxil; CFU, colony-forming unit; CLA, clavulanate; CLOX, cloxacillin; CPR, cefprozil; CTB, ceftibuten; CPD, cefpodoxime; CXM, cefuroxime; D0, CFU/mL on Day zero; DCX, dicloxacin; DF, CFU/mL in drug-free samples on day 5; FET, cefetamet; FLX, flucloxacillin; FPM, faropenem; LEX, cefalexin; MAC, *Mycobacterium avium* complex; PoV, penicillin V; SUP, sulopenem; TBP, tebipenem.

First, we carried out a comprehensive single-point screen of commercially available oral penicillins, cephalosporins, penems, and carbapenems, against 2 reference strains that represent the dominant and most clinically relevant species responsible for MAC-PD, *M. avium* subsp. *hominissuis* MAC109 (MAC109) and *M. intracellulare* ATCC13950 (MI13950). At 10 μM (3.5 to 5.5 µg/mL; Supplementary Table 1), we found that tebipenem and sulopenem—both in phase 3 clinical development—completely inhibited growth of MAC109 and MI13950, with or without a β-lactamase inhibitor (BLI). Several oral penicillins and cephalosporins also showed attractive activity against MI13950 and modest activity against MAC109, with subtle to no dependence on BLI (Figure 1*A*). The most active representatives of each class were selected for dose-response MIC determination, showing that tebipenem and sulopenem were most and similarly active against both MAC109 and MI13950, and that all other β-lactams were more potent against MI13950 than MAC109 (Supplementary Figure 1). A large shift was observed between the MIC_50_ and MIC_90_ (concentrations that inhibit growth by 50% and 90%, respectively), most pronounced for the cephalosporins against MAC109, for which approximately 100-fold differences were measured (Supplementary Table 2 and Supplementary Figure 1). The superior potency against MI13950 was consistent with a targeted screen of 4 parenteral cephalosporins, revealing selective activity against *M. intracellulare* but not *M. avium* [9]. All MIC_50_ values were below the clinical breakpoints published for other pulmonary pathogens, while most MIC_90_ exceeded these breakpoints. These dose-response growth inhibition data also confirmed the weak-to-no dependence on BLI observed in the single-point screen (Supplementary Table 2).

Based on these results, we selected the most potent β-lactam from each class—tebipenem (carbapenem), sulopenem (penem), amoxicillin (penicillin), and cefuroxime (cephalosporin)—for systematic combination studies to identify potential growth inhibition synergies against MAC109 and MI13950. We found that cefuroxime strongly enhanced the growth inhibitory activity of tebipenem, sulopenem, and amoxicillin (fractional inhibitory concentration index of 0.07, 0.19, and 0.31, respectively; Table 1). Interestingly, stronger synergies were seen against MAC109 than MI13950, thus compensating for the weaker activity of single amoxicillin and cefuroxime against MAC109 compared to MI13950. MIC_90_ of single and dual β-lactams remained unchanged in the presence of 4% human serum albumin (Supplementary Table 3), consistent with the generally low plasma protein binding of this class. To determine how these potencies compare to clinically achieved concentrations, we calculated the fraction of the dosing interval during which plasma concentrations are above the MIC_90_ against MAC109 and MI13950, corrected for protein binding, that is T > *f*MIC. We used dosing schedules of published phase 3 trials for tebipenem (600 mg 3 times a day, NCT03788967) and sulopenem (500 mg 2 times a day with adjunctive probenecid to reduce renal clearance, NCT05584657), and doses administered to patients with lower respiratory tract infections for amoxicillin (875 mg 2 times a day [13]). Combined with cefuroxime, sulopenem T > *f*MIC approached 100%, tebipenem T > *f*MIC ranged from 65% to 100%, and amoxicillin T > *f*MIC ranged from 40% to 75% (Figure 1*B*) at their clinical doses. These PK-PD indices hold promise because 40% T > *f*MIC is a common PK-PD target for β-lactams [13].

**Table 1.** Growth Inhibition Synergies of Selected β-lactams Against the *Mycobacteriumavium* Subsp. *hominissuis* and *Mycobacteriumintracellulare* Reference Strains

β-Lactam		Class	*M. avium* Subsp. *hominissuis* MAC109			*M. intracellulare* ATCC 13950		
Drug A	Drug B		MIC_90_, μg/mL^a^		FICI^b^	MIC_90_, μg/mL^a^		FICI
			Alone	Combined		Alone	Combined	
TBP		Carbapenem	0.5	0.13	0.75	0.25	0.13	0.63
	SUP	Penem	0.25	0.13		0.5	0.06	
TBP		Carbapenem	1	0.25	0.5	0.5	0.13	0.5
	AMX	Penicillin	4	1		1	0.25	
TBP		Carbapenem	1	0.007	**0.07**	0.5	0.13	0.5
	CXM	Cephalosporin	32	0.063		1	0.25	
TBP		Carbapenem	1	0.016	**0.08**	0.5	0.13	0.5
	CDN^c^	Cephalosporin	32	2		0.13	0.03	
SUP		Penem	0.5	0.13	0.75	0.13	0.016	0.62
	AMX	Penicillin	4	2		0.25	0.13	
SUP		Penem	0.25	0.03	**0.19**	0.13	0.016	0.62
	CXM	Cephalosporin	32	2		0.5	0.25	
SUP		Penem	0.25	0.03	**0.19**	0.13	0.016	0.62
	CDN	Cephalosporin	32	2		0.063	0.03	
AMX		Penicillin	4	1	**0.31**	2	0.5	0.75
	CXM	Cephalosporin	32	2		2	1	
AMX		Penicillin	4	1	**0.38**	1	0.25	0.5
	CDN	Cephalosporin	32	4		0.5	0.13	

Abbreviations: AMX, amoxicillin; ATCC, American Type Culture Collection; CDN, cefditoren; CXM, cefuroxime; FICI, fractional inhibitory concentration index; MBC_90_, 90% minimum bactericidal concentration; MIC_90_, 90% minimum inhibitory concentration; SUP, sulopenem; TBP, tebipenem.

^a^The standard checkerboard titration assay was used as described previously [8]. Concentration ranges were TBP and SUP, 0.008 to 2 μg/mL; AMX, 0.03 to 16 μg/mL; CXM and CDN, 0.03 to 32 μg/mL.

^b^FICI was calculated using the MIC_90_ of the cultures was observed, as follows: (MIC_A combi_/MIC_A alone_) + (MIC_B combi_/MIC_B alone_). An FICI of < 0.5 was defined as synergy (highlighted in bold). The experiment was carried out twice yielding similar results. *f*T > MIC_90_ and *f*T > MBC_90_ were inferred from clinical pharmacokinetics data in [13, 14, 15].

^c^CDN is shown as it achieved synergies comparable to CXM. However, CXM was selected for subsequent experiments given its higher clinical breakpoint against pulmonary pathogens (Supplementary Table 2).

Next, we selected cefuroxime as the partner drug to test how these results extend to a panel of *M. avium* and *M. intracellulare* reference strains and clinical isolates. Of the 22 strains tested, 15 were susceptible to tebipenem-cefuroxime below the clinical breakpoint of 0.125 μg/mL for tebipenem (Supplementary Table 2), and 11 were susceptible to sulopenem-cefuroxime below the tentative clinical breakpoint of 0.5 μg/mL for sulopenem against complicated urinary tract infection (Supplementary Table 2). Eight isolates were susceptible to amoxicillin-cefuroxime below the breakpoint of 2 μg/mL for amoxicillin established for pulmonary infections. Cefuroxime was kept at a fixed concentration of 1 μg/mL, or 0.1 μg/mL for 4 *M. intracellulare* isolates against which its MIC is lower than 1 μg/mL (Supplementary Table 4). Despite the limited number of clinical isolates surveyed, the MIC_90_ variability was high, as commonly seen for most antibiotics used in the treatment of NTM-PD [5]. To test whether the lower susceptibility of some isolates may be associated with β-lactamase activity, we compared potencies with and without clavulanate across the strain panel, not detecting significant differences (Supplementary Figure 2), suggesting that β-lactamase(s) may not drive intrinsic resistance of these isolates.

To determine whether the synergies in growth inhibition translate into enhanced bactericidal activity, we carried out concentration-kill experiments for the 3 pairs shown in Figure 1*B*, which each exhibit *f*T > MIC_combo_ (that is MIC within the 2-drug combination) of 40% to 100%. As seen in growth inhibitory activity, cefuroxime significantly enhanced the bactericidal activity of tebipenem, sulopenem, and amoxicillin (Supplementary Table 5). Combined with cefuroxime, tebipenem, and sulopenem achieved a 3-log kill (MBC_99.9_) at 0.064 μg/mL (Figure 1*C*), at or below current clinical breakpoint estimates against pulmonary infections. Calculating the time above the MBC_90_ of single and dual β-lactams confirmed that this drug class is bactericidal around the MIC, delivering almost identical T > MIC_90_ and T > MBC_90_ (Figure 1*B*).

## DISCUSSION

We have identified all-oral β-lactam combinations with enhanced bactericidal activity at concentrations achieved in patients for 40% to 100% of the dosing interval. Several important factors contribute to the likelihood that these in vitro observations will extend from bench to bedside. First, β-lactams exhibit largely synchronized PK and tissue distribution patterns, thus optimizing the probability that the pathogen is exposed to matching concentrations on the space-time axes. Second, we have confirmed that synergies in growth inhibition translate into enhanced bactericidal activity. Third, the MIC of the proposed combinations are within the clinical breakpoints established for other pulmonary infections, and the PK-PD targets of *f*T > MIC_90_ > 40% are met for all 3 combinations.

While tebipenem and sulopenem are still in phase 3, the amoxicillin-cefuroxime pair can be tested as salvage therapy in refractory MAC-PD patients in combination with clarithromycin. It achieves concentrations in excess of the MBC_90_ for 30% to 40% of the dosing interval with the standard dosing regimen of 875 mg 2 times a day (Figure 1*B*), a PK-PD target that could be improved with 3 times a day dosing, as used in clinical practice against other bacterial infections. Neurological complications such as β-lactam–induced lowering of the seizure threshold in patients with neurological disorders and renal insufficiency have been observed, which may require monitoring upon administration of dual β-lactams. Despite the limited number of clinical isolates surveyed, the MIC_90_ variability was high, as commonly seen for most antibiotics used in the treatment of NTM-PD [5]. Thus, individualized drug susceptibility testing for these promising β-lactam pairs against each patient's isolate would be required prior to therapy initiation for MAC-PD, potential creating programmatic challenges in some clinical settings.

Combined with cefuroxime, the MBC_99.9_ of tebipenem and sulopenem is at or below 0.064 μg/mL, a concentration that is exceeded for most of the dosing interval, taking plasma protein binding into consideration. In comparison, cefoxitin and imipenem, parenteral β-lactams used in the treatment of *M. abscessus* PD, both require 3 daily infusions, exhibit MIC distributions that center around 8 to 32 μg/mL, and achieve markedly less attractive T > MIC.

## Supplementary Data

Supplementary materials are available at *The Journal of Infectious Diseases* online (http://jid.oxfordjournals.org/). Supplementary materials consist of data provided by the author that are published to benefit the reader. The posted materials are not copyedited. The contents of all supplementary data are the sole responsibility of the authors. Questions or messages regarding errors should be addressed to the author.

## Supplementary Material

jiad591_Supplementary_Data

## Notes

***Acknowledgments.*** We are grateful to Drs Kenneth Olivier, Eric Nuermberger and Drs Steven Aronin and Sailaja Puttagunta from Iterum Therapeutics for stimulating discussion. The authors dedicate this article to the late Won-Jung Koh, who established the Samsung Medical Center nontuberculous mycobacterial cohort from which the clinical isolates used in this study were obtained. Won-Jung Koh passed away in August 2019.

***Author contributions.*** D. A. N. contributed design and execution of all experiments, and visualization. S. J. S., S. -Y. K., and B. W. J. contributed clinical isolates. V. D. drafted the manuscript. T. D. contributed supervision. All authors edited the manuscript.

***Financial support.*** This work was supported by the National Institute of Allergy and Infectious Diseases, National Institutes of Health (grant number R01-AI132374 to T. D. and V. D.); Iterum Therapeutics International, Ltd, a limited liability company incorporated in Ireland with offices at Fitzwilliam Court, 1st Floor, Leeson Close, Dublin 2, D02 YW24, Ireland; and the Center for Discovery and Innovation, a New Jersey nonprofit organization with offices located at 111 Ideation Way, Nutley, NJ 07110, USA. Funding to pay the Open Access publication charges for this article was provided by the Center for Discovery and Innovation, Hackensack Meridian health.
