# Supplementary material for: Oral β-Lactam Pairs for the Treatment of Mycobacterium avium Complex Pulmonary Disease
Source: J Infect Dis. 2023 Dec 27;230(2):e241–6. doi: 10.1093/infdis/jiad591 (PMC11326814; doi:10.1093/infdis/jiad591)
Supplement: jiad591_Supplementary_Data [file jiad591_supplementary_data.docx]

**SUPPLEMENTAL INFORMATION**

Oral β-lactam pairs for the treatment of *Mycobacterium avium* complex pulmonary disease

Dereje A. Negatu ^a, b^, Sung Jae Shin ^c^, Su-Young Kim ^d^, Byung Woo Jhun ^d^, Véronique Dartois ^a, e, #^ and Thomas Dick ^a, e, f^

^a^ Center for Discovery and Innovation, Hackensack Meridian Health, Nutley, New Jersey, USA

^b^ Center for Innovative Drug Development and Therapeutic Trials for Africa (CDT-Africa), Addis Ababa University, Addis Ababa, Ethiopia

^c^ Department of Microbiology, Graduate School of Medical Science, Brain Korea 21 Project, Yonsei University College of Medicine, Seoul, South Korea

^d^ Division of Pulmonary and Critical Care Medicine, Department of Medicine, Samsung Medical Center, Sungkyunkwan University School of Medicine, Seoul, South Korea.

^e^ Department of Medical Sciences, Hackensack Meridian School of Medicine, Nutley, New Jersey, USA

^f^ Department of Microbiology and Immunology, Georgetown University, Washington, DC, USA

**Supplemental Table 1**. Selected features of oral β-lactams used in this study.

| Class | Drug | Abbreviation | Generation | FDA status | MW | 10 μM in μg/mL | Commercial source  (Catalog#) ^[a]^ |
| --- | --- | --- | --- | --- | --- | --- | --- |
| penicillin | ampicillin | AMP |  | Approved | 349.4 | 3.49 | MedChemExpress  (HY-B0522) |
|  | amoxicillin | AMX |  | approved  (NCT02381470 for TB) | 419.45 | 4.19 | Sigma-Aldrich (1031503) |
|  | cloxacillin | CLOX |  | Approved | 475.88 | 4.76 | Sigma-Aldrich (PHR1922) |
|  | dicloxacin | DCX | Second | Approved | 492.31 | 4.92 | Sigma-Aldrich (46182) |
|  | flucloxacillin | FLX |  | Approved | 475.85 | 4.76 | Sigma-Aldrich (SML1023) |
|  | penicillin V | PoV | First | Approved | 388.48 | 3.88 | Sigma-Aldrich (PHR2644) |
|  | amdinocillin | AMD | First | Approved | 325.43 | 3.25 | MuseChem (32887-01-7) |
| cephalosporin | cefetamet | FET | Third | Phase 4 (NCT04664803) | 548.03 | 5.48 | MedChemExpress  (HY-B1894A) |
|  | cefditoren | CDN | Third | Approved | 528.56 | 5.29 | MedChemExpress  (HY-17452) |
|  | cefador | CEC | Second | Approved | 385.82 | 3.86 | Sigma-Aldrich (PHR1283) |
|  | cefadroxil | CFR | First | approved  (NCT02381470 for TB) | 381.4 | 3.81 | Sigma-Aldrich (C0650000) |
|  | cefdinir | CDR | Third | Approved | 395.41 | 3.95 | Sigma-Aldrich (C7118) |
|  | cefixime | CFM | Third | Approved | 453.45 | 4.53 | Sigma-Aldrich (CDS021590) |
|  | cefpodoxime | CPD | Third | Approved | 427.46 | 4.27 | Sigma-Aldrich (32344) |
|  | cefprozil | CPR | Second | Approved | 389.43 | 3.89 | Sigma-Aldrich (Y0001371) |
|  | ceftibuten | CTB | Third | Approved | 410.42 | 4.10 | Sigma-Aldrich (SML0037) |
|  | cefuroxime | CXM | Second | Approved | 446.37 | 4.46 | Sigma-Aldrich (C4417) |
|  | cephalexin | LEX | First | Approved | 347.39 | 3.47 | Sigma-Aldrich (PHR1848) |
|  | cefradine | CED | First | Approved | 349.4 | 3.49 | Sigma-Aldrich (C0690000) |
|  | cefaloglycin | CEG | First | Approved for veterinary use | 405.43 | 4.05 | MuseChem (3577-01-3) |
| penem/ carbapenem | tebipenem | TBP |  | approved in Japan for pediatric use;  Phase 3 (NCT02381470) | 383.49 | 3.83 | MuseChem (161715-21-5) |
|  | sulopenem | SUP |  | Phase 3 (NCT05584657) | 349.45 | 3.49 | Sigma-Aldrich (PZ0042) |
|  | faropenem | FPM |  | Approved in Japan and India (NCT02381470 for TB) | 307.3 | 3.07 | Sigma-Aldrich (F8182) |

^[a]^ Stock solutions of all drugs were prepared in dimethyl sulfoxide (DMSO) except ampicillin, cefaclor and cephalexin which were dissolved in water. MW: molecular weight

**Supplemental Table 2**. Minimum inhibitory concentrations (MIC, μg/mL) of selected β-lactams against the *M. avium* subsp. *hominissuis* and *M. intracellulare* reference strains, and published breakpoints against pulmonary infections.

| β-lactam | β-lactamase inhibitor ^[a]^ | *M. avium* subsp. *hominissuis* MAC 109 ^[b]^ | | | *M. intracellulare* ATCC 13950 ^[b]^ | | | Clinical breakpoint ^[c]^ | |
| --- | --- | --- | --- | --- | --- | --- | --- | --- | --- |
|  |  | MIC_50_ | MIC_90_ | MIC_Vis_ | MIC_50_ | MIC_90_ | MIC_Vis_ | μg/mL | reference |
| TBP | none | **0.1** | 0.5 | 1 | 0.25 | 0.75 | 1 | 0.125 ^[d]^ | [1-3] |
|  | CLA | **0.03** | 0.25 | 1 | **0.12** | 0.5 | 1 |  |  |
|  | AVI | **0.04** | 0.5 | 1 | 0.15 | 0.75 | 1 |  |  |
| SUP | none | **0.02** | **0.2** | 0.5 | **0.05** | 0.5 | 2 | 0.5 ^[e]^ | [4, 5] |
|  | CLA | **0.01** | **0.2** | 0.5 | **0.06** | 0.5 | 2 |  |  |
|  | AVI | **0.04** | **0.2** | 0.5 | **0.05** | 0.5 | 2 |  |  |
| FPM | none | 0.2 | 1.5 | 4 | 0.15 | 0.75 | 2 | Not yet available | n.a. |
|  | CLA | 0.05 | 1 | 4 | 0.06 | 0.5 | 2 |  |  |
|  | AVI | 0.1 | 1 | 4 | 0.1 | 0.75 | 2 |  |  |
| PoV | none | **0.1** | **3.5** | 8 | **0.15** | 2 | 8 | 2 | [6] |
|  | CLA | **0.03** | **1.5** | 8 | **0.15** | 2 | 8 |  |  |
|  | AVI | **0.06** | **1.5** | 8 | **0.13** | **1.5** | 8 |  |  |
| AMX | none | **0.2** | 3 | 8 | **0.2** | **0.5** | 8 | 2 | [6, 7], EUCAST |
|  | CLA | **0.03** | **1.5** | 8 | **0.04** | **0.8** | 8 |  |  |
|  | AVI | **0.03** | 2 | 8 | **0.08** | **1** | 8 |  |  |
| CXM | none | **0.3** | 25 | >32 | **0.25** | 1 | 4 | 1 | [6-9] |
|  | CLA | **0.06** | 25 | >32 | **0.3** | 1 | 4 |  |  |
|  | AVI | **0.2** | 25 | >32 | **0.25** | 1 | 4 |  |  |
| CDN | none | **<0.03** | 25 | >32 | **0.015** | **0.25** | 4 | 0.125-0.5 | [6, 9, 10] |
|  | CLA | **<0.03** | 25 | >32 | **0.01** | 0.5 | 4 |  |  |
|  | AVI | **<0.03** | 25 | >32 | **0.01** | **0.25** | 4 |  |  |
| CPD | none | **0.04** | >32 | >32 | **0.06** | 1.5 | 8 | 0.5 | [6] |
|  | CLA | **0.03** | >32 | >32 | **0.06** | 2 | 8 |  |  |
|  | AVI | **0.04** | >32 | >32 | **0.06** | 1.5 | 8 |  |  |
| CDR | none | **0.4** | >32 | >32 | **0.15** | 0.5 | 4 | 0.5 | [6] |
|  | CLA | **0.06** | >32 | >32 | **0.1** | 0.5 | 4 |  |  |
|  | AVI | **0.25** | >32 | >32 | **0.15** | 0.5 | 4 |  |  |
| RIF | n.a. | **0.01** | **0.03** | **0.13** | **0.04** | **0.06** | **0.13** | 0.5 | [11] |
| RFB |  | **0.03** | **0.08** | 0.13 | **0.08** | 0.2 | 0.5 | 0.125 |  |
| CLR |  | **0.2** | **0.3** | **0..5** | **0.08** | **0.4** | **0.25** | 2 | [12] |
| EMB |  | **0.8** | **6** | 16 | **1.2** | **1.8** | **2** | 8 | [13] |

MICs below the clinical breakpoints are highlighted in bold.

^[a]^ CLA: clavulanic acid at 2.5 μg/mL; AVI: avibactam at 4 μg/mL

^[b]^ *Mycobacterium avium* subsp. *hominissuis* MAC109 was provided by Dr Petros Karakousis (Johns Hopkins University). *M. intracellulare* ATCC 13950 was purchased from the American Type Culture Collection. The MIC of selected β-lactams was determined using the CLSI broth microdilution method substituting CAMHB with complete Middlebrook 7H9 broth (271310; BD Difco, Sparks, MD, USA) supplemented with 0.05% Tween 80, 0.2% glycerol, and 5% oleic acid, albumin, dextrose, and catalase (OADC) enrichment to optimize growth rate and minimize drug incubation time [14, 15] given the limited stability of β-lactams in culture medium [16]. In brief, a 10-point 2-fold serial dilution of compounds was performed in 96-well plates (Costar 3370; Corning, USA) starting at twice the desired highest concentration. Exponentially growing cultures (with an initial OD_600_ of 0.4 to 0.8) were adjusted to 10^5^ CFU/mL in Middlebrook 7H9 broth and seeded onto the 96-well plates containing serially diluted compounds to a final volume of 200 μL/well. The plates were sealed with parafilm, placed onto wet paper towels in a water-tight box, and incubated for 7 days at 37°C with orbital shaking at 110 rpm. The absorbance at 600 nm (OD_600nm_) was measured using a TECAN Infinite Pro 200 plate reader after resuspension. The percent growth inhibition was calculated relative to the untreated controls. MIC values are averages of two biological repeats. Comparing these results with **Figure 1c** highlights that carbapenems are bactericidal around their MIC_90_, as previously described against other pathogens [17]. For example, tebipenem alone achieves both 90% growth inhibition (as measured by OD_600nm_, this table) and 2-log kill (as measured by CFU, **Figure 1c**) at 0.5 μg/mL.

^[c]^ established for other pulmonary infections unless indicated otherwise, retrieved from publications or EUCAST (<https://www.eucast.org/clinical_breakpoints>) and CLSI websites.

^[d]^ tentative breakpoint for uncomplicated urinary tract or bloodstream infections by gram-negative bacteria

^[e]^ breakpoint established for complicated urinary tract infections due to Enterobacterales, personal communication from Dr Steven Aronin, Iterum Therapeutics plc.

Drug abbreviations for β-lactams are listed in Supplemental Table 1. RIF: rifampicin, RBT: rifabutin, CLR: clarithromycin, EMB: ethambutol, n.a.: not applicable

**Supplemental Table 3**. Effect of human serum albumin on MIC of single and dual β-lactams

|  | **MIC_90_** (μg/mL) | | | |
| --- | --- | --- | --- | --- |
|  | MAC109 | | MI 13950 | |
|  | **TBP** | **TBP + CXM** | **TBP** | **TBP + CXM** |
| 7H9 | 0.2 | 0.004 | 0.3 | 0.2 |
| 7H9 with 4% huSA | 0.2 | 0.004 | 0.3 | 0.15 |
|  | **SUP** | **SUP + CXM** | **SUP** | **SUP + CXM** |
| 7H9 | 0.12 | 0.008 | 0.125 | 0.6 |
| 7H9 with 4% huSA | 0.12 | 0.004 | 0.25 | 0.06 |
|  | **AMX** | **AMX + CXM** | **AMX** | **AMX + CXM** |
| 7H9 | 1 | 0.25 | 0.3 | 0.15 |
| 7H9 with 4% huSA | 0.5 | 0.125 | 0.4 | 0.2 |
|  | **OPC-167832** | | | |
| 7H9 | 0.4 | | >32 | |
| 7H9 with 4% huSA | 4 | | >32 | |
|  | **Clarithromycin** | | | |
| 7H9 | 0.25 | | 0.6 | |
| 7H9 with 4% huSA | 0.8 | | 1.5 | |

huSA: human serum albumin; OPC-167832, positive control [22]

**Supplemental Table 4**. Growth inhibitory activity of selected single and dual β-lactams against MAC reference strains and clinical isolates

| ***M. avium* complex reference strain or isolate** ^[a]^ | CLR DST | rrl (2058/2059) | **MIC_90_ (μg/mL)** | | | | | | | | |
| --- | --- | --- | --- | --- | --- | --- | --- | --- | --- | --- | --- |
|  |  |  | CXM alone | TBP | | SUP | | AMX | | CLR | |
|  |  |  |  | Alone | + CXM ^[b]^ | Alone | + CXM | Alone | + CXM | Alone | + CXM |
| *M. avium* subsp *hominissuis* MAC 109 | S | WT | 32 | 0.3 | 0.008 | 0.13 | 0.008 | 3 | 0.5 | 0.25 | 0.15 |
| *M. intracellulare* ATCC 13950 | S | WT | 0.5 | 0.25 | 0.125 | 0.125 | 0.03 | 0.25 | 0.125 | 0.2 | 0.1 |
| *M. chimerae* CCUG 50989 | S | WT | 4 | 0.5 | 0.001 | 0.2 | 0.001 | 1 | 0.03 | 0.1 | 0.02 |
| *M. avium* subsp *avium* ATCC 35717 | S | WT | >32 | 1 | 0.07 | 0.25 | 0.07 | 6 | 3 | 1 | 0.2 |
| *M. avium* Chester ATCC 700898 | S | WT | 32 | 2 | 0.5 | 1.5 | 0.4 | 4 | 1.5 | 0.8 | 0.8 |
| *M. avium* subsp. *hominissuis* 11 | S | WT | 32 | 0.4 | 0.05 | 0.2 | 0.04 | 6 | 2 | 0.2 | 0.12 |
| *M. avium* SC409 | S | WT | 4 | 0.5 | 0.008 | 0.2 | 0.05 | 5 | 2 | 0.4 | 0.25 |
| *M. avium* SC489 | S | WT | 8 | 2 | 0.8 | 1 | 0.25 | 6 | 2.5 | 3 | 3 |
| *M. avium* SC501 | S | WT | 16 | 1 | 0.13 | 0.3 | 0.25 | 16 | 4 | 1 | 1 |
| *M. avium* SC490 | S | WT | 32 | 5 | 3 | 2 | 1 | 6 | 4 | 1 | 0.8 |
| *M. avium* SC422 | R | A/G | >32 | 1.5 | 0.5 | 0.8 | 0.13 | 16 | 4 | >32 | >32 |
| *M. avium* SC498 | R | A/C | 16 | 1.5 | 0.06 | 0.5 | 0.06 | 8 | 4 | >32 | >32 |
| *M. avium* SC397 | R | A/C | 4 | 3 | 1.5 | 0.5 | 0.25 | 5 | 3 | >32 | >32 |
| *M. avium* SC406 | R | G/A | 4 | 1.5 | 0.02 | 0.5 | 0.03 | 3 | 1 | >32 | >32 |
| *M. intracellulare* SC491 | S | WT | 32 | 5 | 2 | 4 | 3 | 10 | 6 | 1 | 0.8 |
| *M. intracellulare* SC502 | S | WT | 0.25 | 0.125 | 0.0156 | 0.0625 | 0.0156 | 0.125 | 0.0625 | 0.13 | 0.13 |
| *M. intracellulare* SC495 | S | WT | 32 | 4 | 3 | 4 | 3 | 16 | 16 | 0.4 | 0.3 |
| *M. intracellulare* SC497 | S | WT | 0.25 | 0.25 | 0.03 | 0.03 | 0.003 | 0.125 | 0.0625 | 0.2 | 0.2 |
| *M. intracellulare* SC400 | R | C/A | >32 | 1 | 0.5 | 2 | 1 | 6 | 4 | >32 | >32 |
| *M. intracellulare* SC425 | R | C/A | 16 | 2.5 | 2.5 | 2.5 | 1.5 | 10 | 8 | >32 | >32 |
| *M. intracellulare* SC404 | R | A/G | 8 | 4 | 4 | 2 | 1 | 10 | 7 | >32 | >32 |
| *M. intracellulare* SC410 | R | A/T | 0.5 | 0.25 | 0.125 | 0.125 | 0.0625 | 0.5 | 0.25 | 2 | 2 |

^[a]^ *M. avium* subsp. *avium* ATCC 35717, *M. avium* Chester ATCC 700898, and *M. intracellulare* ATCC 13950 were purchased from the American Type Culture Collection. *M. chimerae* CCUG 50989 was purchased from the Culture Collection University of Goteborg. *M. avium* subsp. *hominissuis* MAC109 and *M. avium* subsp. *hominissuis* strain 11 were provided by Dr Petros Karakousis (Johns Hopkins University) and Dr Jung-Yien Chien and Po-Ren Hsueh (National Taiwan University Hospital, Taipei), respectively. The remaining clinical isolates were obtained from Dr Sung Jae Shin (Department of Microbiology, Yonsei University College of Medicine, Seoul, South Korea) and Dr Won-Jung Koh (Division of Pulmonary and Critical Care Medicine, Samsung Medical Center, Seoul, South Korea) [23]. CLR was included in all MIC experiments as a positive control and to monitor assay reproducibility.

^[b]^ Cefuroxime (CXM) was added at a fixed subinhibitory concentration of 1 μg/mL except against strains highlighted in green where the concentration was reduced to the subinhibitory concentration of 0.1 μg/mL since its MIC when used alone was < 1 μg/mL.

**Supplemental Table 5**. Bactericidal activity of single and dual β-lactams against the *M. avium* subsp. *hominissuis* and *M. intracellulare* reference strains.

|  | CXM | TBP | | SUP | | AMX | |
| --- | --- | --- | --- | --- | --- | --- | --- |
|  |  | - CXM | + CXM ^(a)^ | - CXM | + CXM | - CXM | + CXM |
| MAC109 ^(b)^ | | | | | | | |
| MBC_90_ (mg/L) | 16 | 1 | 0.008 | 1 | 0.016 | 8 | 2 |
| MBC_99_ (mg/L) | 32 | 4 | 0.016 | 2 | 0.032 | 16 | 4 |
| MBC_99.9_ (mg/L) | >32 | 4 | 0.064 | 8 | 0.064 | >32 | 8 |
| MI13950 | | | | | | | |
| MBC_90_ (mg/L) | 0.5 | 0.5 | 0.008 | 1 | 0.008 | 2 | 0.5 |
| MBC_99_ (mg/L) | 2 | 1 | 0.016 | 2 | 0.032 | 4 | 1 |
| MBC_99.9_ (mg/L) | 16 | 4 | 0.032 | 8 | 0.064 | >32 | 8 |

^(a)^ CXM at 1 mg/L against MAC109 and at 0.25 mg/L against MI13950

^(b)^ *f*T>MBC_90_ were inferred from clinical PK data provided in [18, 19] and [20].

Supplemental Figures

**Figure S1**. Dose-response growth inhibition curves of selected β-lactams against the reference strains *M. avium* subsp *hominissuis* MAC109 (black symbols) and *M. intracellulare* ATCC 13950 (MI13950, red symbols). Exponentially growing cultures of MAC109 and MI13950 were adjusted to a final bacterial density of approximately 10^5^ CFU/mL and exposed to increasing concentrations of drugs. The optical density at 600 nm (OD_600_) was measured after 7 days of incubation in 96-well plates. These plots help visualize the marked disconnect between MIC_50_ and MIC_90_ for the four cephalosporins against MAC109. A wide shift was also observed, albeit to a lower extent, for the two penicillins against MAC109. Experiments were carried out twice independently and representative graphs are shown (GraphPad Prism 8 software). Average MIC values of both experiments are shown in Supplemental Table 2.

**Figure S2**. Effect of β-lactamase inhibitor clavulanic acid at a fixed concentration of 2.5 μg/mL on the growth inhibitory activity of the four selected β-lactams: tebipenem (TBP), sulopenem (SUP), amoxicillin (AMX) and cefuroxime (CXM) against the complete panel of reference strains and clinical isolates. Each β-lactam was tested at three concentrations centered around their respective clinical breakpoint.

**REFERENCES**

1. Ranasinghe A, Henderson A, Cottrell K, et al. Determining the in vitro susceptibility of tebipenem, an oral carbapenem, against third-generation cephalosporin-resistant Escherichia coli and Klebsiella pneumoniae isolated from bloodstream infections. JAC Antimicrob Resist **2022**; 4(5): dlac105.

2. Critchley IA, Cotroneo NS, Pucci MJ, Jain A, Medes RE. Tebipenem: an oral carbapenem with activity against multi-drug resistant urinary tract infection isolates of Escherichia coli collected from US medical centers during 2019. Open Forum Infect Dis **2020**; 7: S831.

3. Gerges B, Rosenblatt J, Hachem RY, Raad II, Chaftari A-M. In vitro activity of tebipenem against clinically significant gram-negative bacteria isolated from patients with cancer. Open Forum Infect Dis **2021**; 8: S627.

4. Zhanel GG, Pozdirca M, Golden AR, et al. Sulopenem: An Intravenous and Oral Penem for the Treatment of Urinary Tract Infections Due to Multidrug-Resistant Bacteria. Drugs **2022**; 82(5): 533-57.

5. Dunne MW, Aronin SI, Das AF, et al. Sulopenem for the Treatment of Complicated Urinary Tract Infections Including Pyelonephritis: A Phase 3, Randomized Trial. Clin Infect Dis **2023**; 76(1): 78-88.

6. Murphy ME, Powell E, Courter J, Mortensen JE. Predicting Oral Beta-lactam susceptibilities against Streptococcus pneumoniae. BMC Infect Dis **2021**; 21(1): 679.

7. Zafar A, Hasan R, Nizamuddin S, et al. Antibiotic susceptibility in Streptococcus pneumoniae, Haemophilus influenzae and Streptococcus pyogenes in Pakistan: a review of results from the Survey of Antibiotic Resistance (SOAR) 2002-15. J Antimicrob Chemother **2016**; 71 Suppl 1(Suppl 1): i103-9.

8. Al-Waili BR, Al-Thawadi S, Hajjar SA. Impact of the revised penicillin susceptibility breakpoints for Streptococcus pneumoniae on antimicrobial resistance rates of meningeal and non-meningeal pneumococcal strains. Ann Saudi Med **2013**; 33(2): 111-5.

9. Gimenez MJ, Aguilar L, Granizo JJ. Revisiting cefditoren for the treatment of community-acquired infections caused by human-adapted respiratory pathogens in adults. Multidiscip Respir Med **2018**; 13: 40.

10. Lodise TP, Kinzig-Schippers M, Drusano GL, et al. Use of population pharmacokinetic modeling and Monte Carlo simulation to describe the pharmacodynamic profile of cefditoren in plasma and epithelial lining fluid. Antimicrob Agents Chemother **2008**; 52(6): 1945-51.

11. WHO. Technical Report on critical concentrations for drug susceptibility testing of isoniazid and the rifamycins (rifampicin, rifabutin and rifapentine). Geneva: WHO, **2021**.

12. CLSI. Susceptibility Testing of Mycobacteria, Nocardiae, and Other Aerobic Actinomycetes; Approved Standard—Second Edition, **2011**.

13. Schon T, Chryssanthou E. Minimum inhibitory concentration distributions for Mycobacterium avium complex-towards evidence-based susceptibility breakpoints. Int J Infect Dis **2017**; 55: 122-4.

14. Jaffre J, Aubry A, Maitre T, et al. Rational Choice of Antibiotics and Media for Mycobacterium avium Complex Drug Susceptibility Testing. Front Microbiol **2020**; 11: 81.

15. CLSI. Susceptibility testing of Mycobacteria, Nocardia spp., and Other Aerobic Actinomycetes. Wayne, PA: Clinical and Laboratory Standards Institute, **2018**.

16. Negatu DA, Zimmerman MD, Dartois V, Dick T. Strongly Bactericidal All-Oral beta-Lactam Combinations for the Treatment of Mycobacterium abscessus Lung Disease. Antimicrob Agents Chemother **2022**; 66(9): e0079022.

17. Herrera-Espejo S, Del Barrio-Tofino E, Cebrero-Cangueiro T, et al. Carbapenem Combinations for Infections Caused by Carbapenemase-Producing Pseudomonas aeruginosa: Experimental In Vitro and In Vivo Analysis. Antibiotics (Basel) **2022**; 11(9).

18. de Velde F, de Winter BC, Koch BC, van Gelder T, Mouton JW, consortium C-N. Non-linear absorption pharmacokinetics of amoxicillin: consequences for dosing regimens and clinical breakpoints. J Antimicrob Chemother **2016**; 71(10): 2909-17.

19. Eckburg PB, Jain A, Walpole S, et al. Safety, Pharmacokinetics, and Food Effect of Tebipenem Pivoxil Hydrobromide after Single and Multiple Ascending Oral Doses in Healthy Adult Subjects. Antimicrob Agents Chemother **2019**; 63(9).

20. Dunne M, Dunzo E, Puttagunta S. A phase 1 study to assess the pharmacokinetics of sulopenem etzadroxil (PF-03709270). In: IDWeek 2017. San Diego, CA, 2017.

21. Sevillano D, Gimenez MJ, Alou L, et al. Effects of human albumin and serum on the in vitro bactericidal activity of cefditoren against penicillin-resistant Streptococcus pneumoniae. J Antimicrob Chemother **2007**; 60(1): 156-8.

22. Robertson GT, Ramey ME, Massoudi LM, et al. Comparative Analysis of Pharmacodynamics in the C3HeB/FeJ Mouse Tuberculosis Model for DprE1 Inhibitors TBA-7371, PBTZ169, and OPC-167832. Antimicrob Agents Chemother **2021**; 65(11): e0058321.

23. Wang HY, Kim H, Kim S, Kim DK, Cho SN, Lee H. Performance of a real-time PCR assay for the rapid identification of Mycobacterium species. J Microbiol **2015**; 53(1): 38-46.
